# A New Species from *Athous* (*Orthathous*) *acutangulus* Species Group from Turkey

**DOI:** 10.1673/031.012.13001

**Published:** 2012-11-10

**Authors:** Mahmut Kabalak, Osman Sert

**Affiliations:** Hacettepe University Faculty of Science, Department of Biology Section of Applied Biology Beytepe, Ankara, Turkey

**Keywords:** aedeagi, description, differential diagnosis, identification key, morphology

## Abstract

A new Elateridae species, ***Athous* (*Orthathous*) *cagatayae* n. sp.,** is presented from Ankara, Turkey. The morphology of the new species is described. Photographs of imago and aedeagus, aedeagi drawings of the new species, and identification key are given. The new species is discussed with species of ***acutangulus*** group, with a differential diagnosis.

There have been many studies on the subgenus ***Orthathous*** Reitter 1905, which is well represented in Turkey with 46 species, and it has been recorded by various researchers (Mertlik and Platia 2008; Kabalak and Sert 2010; Platia 2010; Platia and Nemeth 2011). ***Athous (Orthathous) cagatayae* n. sp.** belongs to the ***Athous (Orthathous)*** ***acutangulus*** group of the subgenus ***Orthathous***. ***Acutangulus***-group **(*A.* (*O*.)** ***acutangulus*** Fairmaire 1866, ***A.* (*O.*)** ***freudei*** Platia 1989, ***A*. (*O*.)** ***frontalis*** Platia and Schimmel 1991, ***A.* (*O*.) *graecus*** Platia 1989, ***A.* (*O*.) *kabalaki*** Platia 2010, and ***A.* (*O*.)** ***zbuzeki*** Platia and Gudenzi 2007) is separated from other species by a regularly decreasing length of the second, third, and fourth tarsal segments. The new species was given erroneously as ***Athous (O.) carpathicus*** by Kabalak (2004) and Kabalak and Sert (2005). After receiving a paratype, specimens were reexamined and detected as a new species.

## Materials and Methods

The body lengths of the specimens were measured along the midline, from the anterior margin of the frons to the apex of elytra. The widths of the specimens were measured across the broadest part of the elytra. Photographs of imago and aedeagus were taken.

The aedeagus of the new species was drawn in detail, and the aedeagi of other species in the group were re-drawn from literature, except for ***A.* (*O*.) *acutangulus***, which lacks an aedeagus, and ***A. *(*O*.) *kabalaki***, which had an asymmetrical photograph. An identification key of species in the group was prepared as an addition to the identification key of Turkish ***Orthathous*** species, which was given by Platia and Gudenzi (1996). The new species was compared with ***A. *(*O*.) *graecus***, which is close to the new species in the identification key. All species of ***acutangulus***-group are compared by using aedeagi morphologies, collecting months, collecting localities of Turkey, and zoogeographical distributions in Table 1, except for ***A.* (*O*.) *acutangulus***.

**Figure 1.**
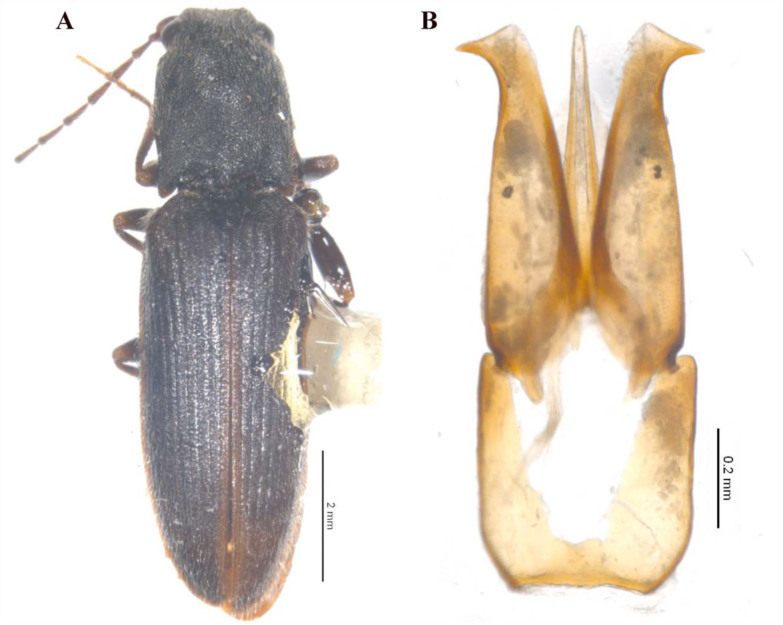
(A) ***Athous* (*Orthathous*) *cagatayae* n. sp.** habitus (scale = 2 mm). (B) Aedeagus habitus (scale = 0.2 mm). High quality figures are available online.

**Figure 2.**
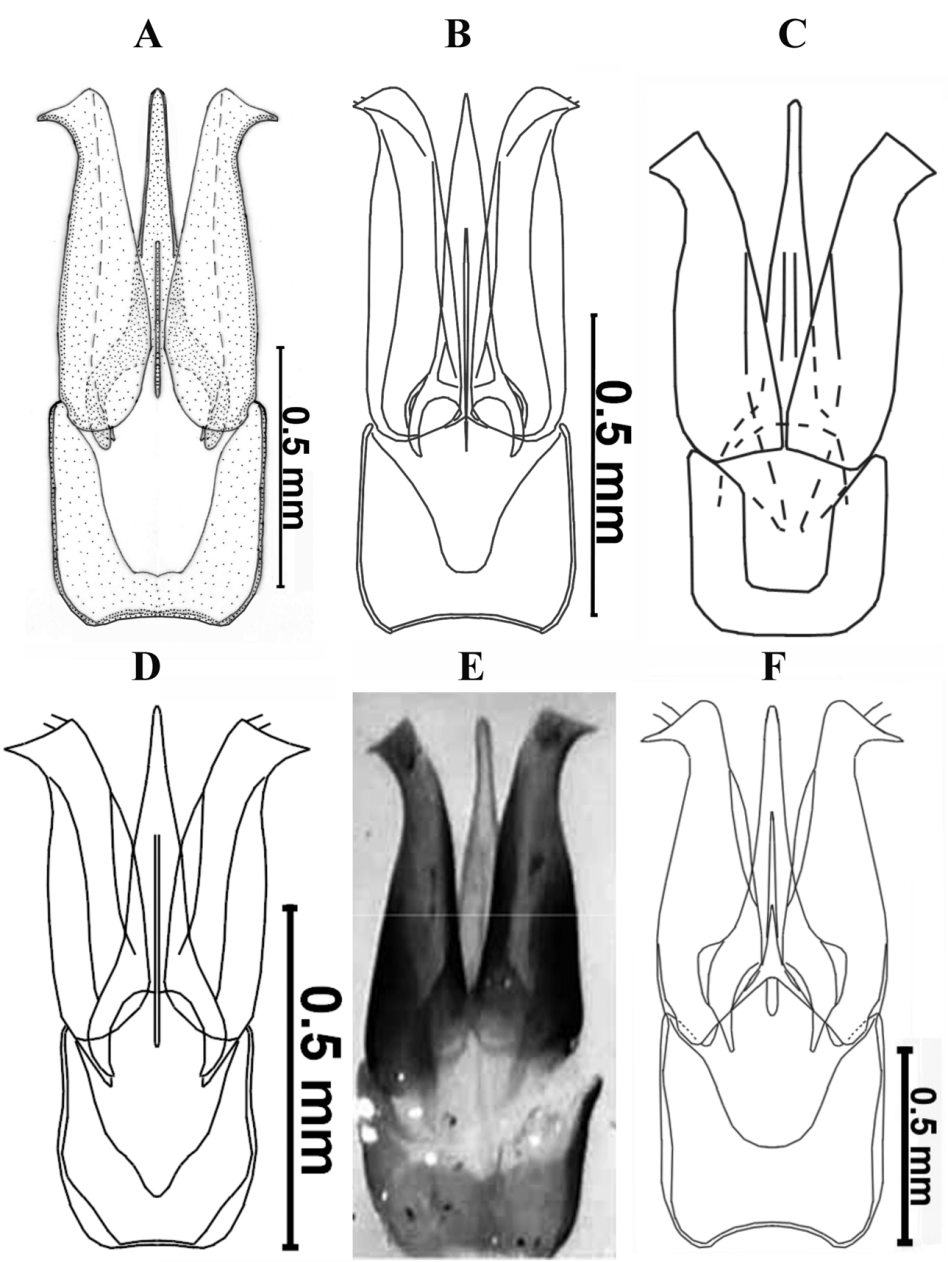
(A) ***Athous(Orthathous)cagatayae* n. sp..** (B) ***Athous (Orthathous) freudei*** (redrawn from Platia 1989). (C) ***Athous (Orthathous) frontalis*** (redrawn from Platia and Schimmel 1991). (D) ***Athous (Orthathous) graecus*** (redrawn from Platia 1989). (E) ***Athous (Orthathous) kabalaki*** (taken from Platia 2010). (F) ***Athous (Orthathous)zbuzeki*** (redrawn from Platia and Schimmel 2007). High quality figures are available online.

***Athous* (*Orthathous*) *cagatayae* Kabalak and Sert, new species (Figures 1, 2a)**
**Type locality: Holotype:** One male from the Ankara province, Çubuk county, between Özlüce and Ovacık villages, 40° 18′ 54″ N, 32° 55′ 46″ E, 1072 m.a.s.l., 25 June 2003, leg. M. Kabalak.
**Paratype:** One male from the Ankara province, Çubuk County, Ömürdede, 30 June 1980, leg. Y. Özdemir. Holotype and paratype are deposited in Hacettepe University Zoology Museum (HUZOM) at Hacettepe University Biology Department, Ankara.
**Holotype:** Male, length 9.56 mm; width 2.57 mm; body dark-brown colored, except for reddish brown antennae, elytral suture, and legs; body covered with slightly long and dense yellowish hairs.
  Head, including eyes, as wide as anterior margin of pronotum, and covered with dense umbilicate punctures, with an impression beginning at the vertex and extending to the fronto-clypeal suture; fronto-clypeal suture distinctly convex, without touching the clypeus.
  Antenna exceed the apices of the posterior angles of pronotum by about four segments; second segment sub-conical, almost as long as it was wide; third segment triangular, 1.9 times longer than the second, and 1.24 times shorter than fourth; second and third taken together clearly longer than fourth; segments four through seven triangular.
  Pronotum 1.13 times longer than wide, slightly convex on the disk, with weakly distinct median carina, sides feebly arcuate, posterior angles slightly divergent, not carinate, apex slightly pointed, and lateral margin fully visible in dorsal view; punctuation generally deep, dense, and umbilicate.
  Scutellum narrower than the inter-elytral space, longer than wide, convex, deep, and scattered punctuated.
  Elytra 2.9 times longer than pronotum, 2.5 times longer than wide, sides sub-parallel from proximal to medio-distal and then gradually narrowing towards apex; striae regularly and indistinctly punctured, interstriae feebly convex, coarsely and simple punctured, with rough surface.
  Legs with second, third, and fourth tarsal segments nearly regularly decreasing in length; fourth tarsal segment small, in dorsal view as long as half of the third, and as long as one-third of fifth segment. Aedeagus length 1.15mm, typical morphology for the genus (Figures 1b and 2a), parameres acutely dentate, and apex slightly angled.
**Female:** Unknown.
**Paratype:** Length 9.41 mm, width 2.74 mm
**Etymology:** The new species is dedicated to emeritus entomologist Prof. Dr. Neçe Çağatay, who made invaluable contributions for development of Entomology Science in Hacettepe University and Turkey.
**Habitat:** The holotype was collected, using an insect net, from herbaceous plants under ***Salix*** sp. along a stream in June 2003.

**Table 1.**
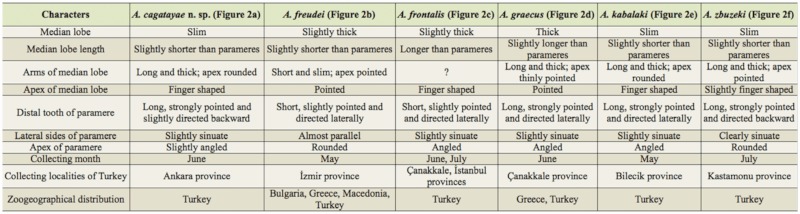
Aedeagi morphologies, collecting months, collecting localities and Zoogeographical distribution comparisons of species **of *Athous* (*O*.) *acutangulus*** group.

## Discussion

***A.* (*O*.) *cagatayae* n. sp.** is close to ***A.* (*O*.)** *graecus,* however it could be separated by the following characters: ***A.* (*O*.) *cagatayae* n. sp.** is larger than ***A.* (*O*.) *graecus*;** elytral suture of the new species is reddish-brown, while it is not reddish-brown in ***A.*** (*O*.) *graecus.*

Aedeagi morphologies of all species were compared (Table 1). First of all, species can be examined in two groups based on length of median lobe. In the first group, the median lobe is longer than the parameres (*4.* (*O.*) ***frontalis*** and ***A.* (*O*.)** *graecus*), while the median lobe is shorter than the parameres in the second group **(*A.* (*O*.) *cagatayae* n. sp., *A.*** **(*O.*) *freudei***, ***A.* (*O*.) *kabalaki***, and ***A.* (*O*.) *zbuzeki*)**. In the first group, ***A.* (*O*.) *frontalis*** and ***A.* (*O.*) *graecus*** can be separated based on the distal tooth of the paramere, which is short and slightly pointed in ***A.* (*O.*) *frontalis***, while it is long and strongly pointed in ***A.* (*O.*) *graecus***. In the second group, ***A.* (*O.*) *freudei*** is separated from ***A.* (*O.*) *cagatayae* n. sp.**, ***A. A.* (*O.*) *kabalaki***, and ***A.* (*O.*) *zbuzeki*** with a short and slightly pointed distal tooth of the paramere. The pointed apex of the median lobe and the rounded apex of the paramere distinguishes ***A.* (*O.*) *zbuzeki*** from ***A.* (*O.*) *cagatayae* n. sp.** and ***A.* (*O.*) *kabalaki*.** According to the direction of the distal tooth of the paramere, and the apex of the median lobe, ***A.* (*O.*)** ***cagatayae* n. sp.** could be differentiated from ***A.* (*O.*) *kabalaki***. ***A.* (*O.*) *cagatayae* n. sp.** has a slightly backward-directed paramere tooth, and a feebly angled apex of the median lobe, while ***A.* (*O.*) *kabalaki*** has a laterally-directed paramere tooth, and an angled apex of the median lobe.

According to literature (Platia 1989; Platia and Schimmel 1991; Platia and Gudenzi 2000, 2007; Platia 2010; Platia and Nemeth 2011), collecting months, collecting localities of Turkey, and zoogeographical distributions of species of ***acutangulus***-group are given (Table 1). Species are present in nature from May to July. Only ***A.* (*O.*) *frontalis*** was collected in two provinces (Çanakkale and Istanbul). ***A.* (*O*.) *freudei***, which is the most common species of ***acutangulus***-group, is distributed throughout Bulgaria, Greece, Macedonia, and Turkey.

**Identification Key to Turkish species of the subgenus *Orthathous* (Platia and Gudenzi 1996) (Males) (Modified from Platia and Gudenzi 1996)**

**1.** Fourth tarsal segment much smaller than third **2**

1′. Second, third, fourth tarsal segments decreasing regularly in length 17 (***acutangulus***-group)

**2.** Larger size on average (length 10.5–12.5 mm; width 2.9–3 mm) **3**

**2′.** Smaller average size (length 6–10 mm; width 2–2.8 mm) **4**

**3.** More robust and longer antennae extending about 4 segments past posterior angles of pronotum ***anatolicus*** Guglielmi and Platia 1985

**3′.** Slimmer and shorter antennae extending only 2.5–3 segments past posterior angles of pronotum ***daccordii*** Guglielmi and Platia 1985

**4.** Viewed dorsally third tarsal segment not dilated, slightly broader apically than fourth **5**

**4′.** Viewed dorsally third tarsal segment dilated, clearly broader apically than fourth **7**

**5.** Third antennal segment two or more times longer than second **6**

**5′.** Third antennal segment less than two times longer than second ***zanettii*** Guglielmi and Platia 1985

**6.** Body larger (length 9–10 mm; width 2.4–2.5 mm); pronotal punctures on disk deeper, simple to feebly umbilicate ***propinquus*** Buysson 1889

**6′.** Body smaller (length 7.5–8 mm; width 2–2.1 mm); pronotal punctures on disk more superficial, clearly umbilicate ***senaci*** Buysson 1889

**7.** Third antennal segment two to more times longer than second **8**

**7′.** Third antennal segment less than two times longer than second **10**

**8.** Shorter antennae extending 2–3 segments past posterior angles of pronotum **9**

**8′.** Longer antennae extending about 4 segments past posterior angles of pronotum ***wewalkai*** Platia 1989

**9.** Body stouter; elytra on the average 2.6–2.7 times longer than pronotum; third antennal segment only two times longer than second ***barriesi*** Platia and Gudenzi 1996

**9.** Body slimmer; elytra on the average 3 times longer than pronotum; third antennal segment more than two times longer than second ***margheritae*** Guglielmi and Platia 1985

**10.** Shorter antennae extending **1–1.5** segments past posterior angles of pronotum **11**

**10′.** Longer antennae extending 2.5–3 segments past posterior angles of pronotum 12

**11.** Body slimmer; elytra 3 times longer than pronotum, with subparallel sides; frons with strong triangular depression ***giannassoi*** Platia and Gudenzi 1996

**11′.** Body stouter; elytra 2.6 times longer than pronotum, sides feebly dilated; frons with shallow depression ***ruffoi*** Guglielmi and Platia 1985

**12.** Head, including eyes, clearly narrower than anterior part of pronotum **13**

**12′.** Head, including eyes, as wide as anterior margin of pronotum **15**

**13.** Frons with deep depression from vertex **14**

**13′.** Frons with depression only near anterior margin ***tribertii*** Guglielmi and Platia 1985

**14.** Longer antennae extending 3 segments past posterior angles of pronotum; elytra as wide as pronotum, with subparallel sides ***audisioi*** Guglielmi and Platia 1985

**14′.** Shorter antennae extending 2.5 segments past posterior angles of pronotum; elytra broader than pronotum, and feebly dilated behind the middle ***lassallei*** Platia and Gudenzi 1996

**15.** Elytra shorter, 2.6–2.9 times longer than pronotum **16**

**15′.** Elytra longer, 3–3.2 times longer than pronotum ***sabatinettii*** Guglielmi and Platia 1985

**16.** Pronotum with a trace of a median longitudinal carina *gudenzii* Guglielmi and Platia 1985

**16′.** Pronotum with a trace of a very narrow and superficial median longitudinal depression ***dasycerus*** Buysson 1890

**17.** Pronotal punctures clearly umbilicate **18**

**17′.** Pronotal punctures not or vaguely umbilicate **20**

**18.** Frons deeply impressed ***frontalis*** Platia and Schimmel 1991

**18′.** Frons slightly or moderately impressed **19**

**19.** Pronotum as long as wide, antennae extending 3 segments past posterior angles of pronotum, and third antennal segment two times longer than second ***zbuzeki*** Platia and Gudenzi 2007

**19′.** Pronotum 1.1 times longer than wide, antennae extending 1.5–2.5 segments past posterior angles of pronotum, and third antennal segment slightly longer than second ***freudei*** Platia 1989

**20.** Frons deeply impressed **21**

**20′.** Frons shallowly impressed **22**

**21.** Body larger (length 9.41–9.56 mm; width 2.57–2.74 mm), elytral suture reddish-brown ***cagatayae* n. sp.**

**21′.** Body smaller (length 7.3 mm; width 1.8 mm), elytral suture not reddish-brown ***graecus*** Platia 1989

**22.** Body longer (length 8–11 mm); pronotum longer than wide ***acutangulus*** Fairmaire 1866

**22′.** Body shorter (length 7.3 mm); pronotum as long as wide ***kabalaki*** Platia 2010
